# Prime Editor Gene Therapy and *TREX1* Mosaicism in Retinal Vasculopathy with Cerebral Leukoencephalopathy

**DOI:** 10.1007/s10875-024-01846-y

**Published:** 2024-12-13

**Authors:** Samuel D. Chauvin, Joe A. Holley, Subhajit Poddar, Cathrine A. Miner, Lindsay Kumble, Jiayuan Fu, Hanka Laue-Gizzi, Todd A. Hardy, Jonathan J. Miner

**Affiliations:** 1 Division of Rheumatology, Department of Medicine, University of Pennsylvania Perelman School of Medicine, Philadelphia, PA USA; 2 RVCL Research Center, University of Pennsylvania Perelman School of Medicine, Philadelphia, PA USA; 3 Faculty of Medicine, University of New South Wales, Sydney, Australia; 4 Department of Neurology, Prince of Wales Hospital, Sydney, Australia; 5 Multiple Sclerosis and Neuroimmunology Clinics, Department of Neurology, Concord Repatriation General Hospital, University of Sydney, Sydney, NSW Australia; 6 Brain & Mind Centre, University of Sydney, Sydney, NSW Australia; 7 Department of Microbiology, University of Pennsylvania Perelman School of Medicine, Philadelphia, PA USA; 8 Institute for Immunology and Immune Health, University of Pennsylvania Perelman School of Medicine, Philadelphia, PA 19104 USA; 9 Colton Center for Autoimmunity, University of Pennsylvania Perelman School of Medicine, Philadelphia, PA 19104 USA

**Keywords:** TREX1, Small vessel disease, Dementia, Vasculopathy, Retinal vasculopathy with cerebral leukoencephalopathy and systemic manifestations, RVCL, RVCL-S, HERNS, Cerebroretinal vasculopathy, CRV, Gene therapy, Prime editor

## Abstract

*TREX1* mutations underlie a variety of human diseases, including retinal vasculopathy with cerebral leukoencephalopathy (RVCL or RVCL-S), a catastrophic adult-onset vasculopathy that is often confused with multiple sclerosis, systemic vasculitis, or systemic lupus erythematosus. Patients with RVCL develop brain, retinal, liver, and kidney disease around age 35–55, leading to premature death in 100% of patients expressing an autosomal dominant C-terminally truncated form of TREX1. We previously demonstrated that RVCL is characterized by high levels of DNA damage, premature cellular senescence, and risk of early-onset breast cancer before age 45. Here, we report human *TREX1* mosaicism causing organ-limited RVCL in the retina, as well as a gene therapy to synthetically create *TREX1* mosaicism as a potential treatment for RVCL. In our patient with organ-limited disease, the mosaic *TREX1* mutant allele underwent germline transmission to 3 children, who developed severe multi-organ disease at ~ age 40, unlike their mosaic parent, who has organ-limited disease at age 74. Additionally, we describe our *TREX1* prime editor gene therapy that corrects the most common RVCL-causing *TREX1* variant in cell culture and in mice. Thus, *TREX1* mosaicism causes organ-limited RVCL with a normal lifespan, suggesting that a gene therapy to create *TREX1* mosaicism in adults may someday become useful as a treatment for patients with RVCL.

## Introduction

*TREX1* genetic variants cause unique disease phenotypes depending on whether TREX1 localization or enzymatic activity is affected. Whereas catalytic domain mutations in *TREX1* cause autosomal recessive Aicardi-Goutières syndrome (AGS) and autosomal dominant chilblain lupus, C-terminal truncations distal to the exonuclease region cause a completely different disease known as retinal vasculopathy with cerebral leukoencephalopathy (RVCL or RVCL-S; previously known as HERNS or CRV) [1–4]. Although somatic mosaicism has been reported for some monogenic autoimmune and autoinflammatory diseases, *TREX1* mosaicism had not previously been described in RVCL. Here, we report *TREX1* mosaicism causing organ-limited maternal RVCL, as well as inherited multi-organ disease in her 3 children. Unlike the premature death observed universally with germline RVCL-causing *TREX1* mutations, we find that mosaicism is associated with a normal lifespan.

TREX1 is normally anchored at the endoplasmic reticulum (ER) membrane and excluded from the nucleus [3]. However, in patients with RVCL, a mislocalized but active TREX1 3’-5’ DNA exonuclease constitutively enters the nucleus and damages genomic DNA, leading to a syndrome of DNA damage, endotheliopathy, and inflamm-aging [5]. Indeed, cells expressing C-terminally truncated, enzymatically active TREX1 undergo rapid senescence in cell culture, but without inducing a significant type I interferon (IFN) response under most conditions [5].

Historically, RVCL has been thought to cause premature death in 100% of affected individuals [6]. Gene therapies and personalized medicines for RVCL are currently in clinical development, including a gene therapy developed by our laboratory [7], but the target cells for *TREX1* gene therapies remain to be fully defined. Since gene therapies create synthetic mosaicism in adult animals, defining the clinical phenotypes of naturally occurring *TREX1* mosaicism would have major implications for understanding the potential benefits of *TREX1*-targeted gene therapies.

RVCL is often confused with multiple sclerosis because of brain lesions, and with systemic lupus erythematosus because of autoantibodies, kidney damage, and Raynaud’s syndrome. Patients with RVCL also experience vision loss due to retinal vascular disease, as well as brain necrosis, renal failure, osteonecrosis, and liver injury [6]. Patients with RVCL are usually healthy until around ages 35–55 years, when they begin to develop severe multi-organ vasculopathy leading to disability and premature death in 100% of patients [6]. There is currently no effective treatment for RVCL / RVCL-S.

It is very unusual for a patient with RVCL to live beyond the 6th or 7th decades of life, and many succumb to disease before age 50 [6]. Here, we show that maternal *TREX1* mosaicism is associated with the longest lifespan of any reported patient with RVCL, well into the 8th decade of life. Our patient with *TREX1* mosaicism exhibits near-complete sparing of all the usually affected organs, with the exception of the retina. However, germline transmission to 3 of her children led to significant, multi-organ disease in her progeny by around age 40. We also report our invention of a CRISPR/Cas9 prime editor gene therapy that we developed for RVCL. Since *TREX1* mosaicism prolongs the lifespan and results in organ-specific disease, our discoveries may provide hope that tissue- and cell type-specific correction of a *TREX1* variant might become useful to limit RVCL-associated pathology and to prolong life.

## Results

**Fig. 1:**
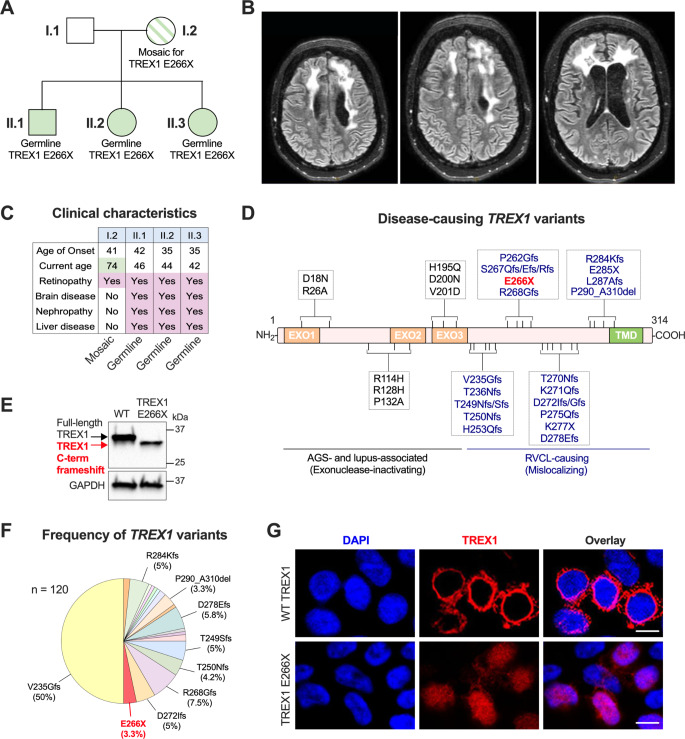
Organ-limited RVCL due to parental *TREX1* mosaicism with classic multi-organ RVCL in her progeny. (**A**) Pedigree of the family with inheritance of a mosaic disease-causing *TREX1* mutation. Shaded boxes represent patients with germline TREX1 E266X mutations and the patterned shading indicates mosaicism in the mother. (**B**) MRI of patient II.2 from (**A**) with inherited RVCL. (**C**) Key clinical features in the affected family. (**D**) Schematic of the TREX1 protein with known disease-causing mutations. (**E**) Representative Western blot of TREX1 E266X expression in 293T cells. Data in (**E**) are representative of *n* = 3 independent experiments. (**F**) Frequency of TREX1 protein variants found in our cohort of 120 patients with RVCL. (**G**) Representative confocal immunofluorescence images of 293T cells expressing HA-tagged WT TREX1 or TREX1 E266X with immunostaining for TREX1 (HA). Scale bar = 15 μm. Data are representative of all cells observed in 2 independent experiments

We identified 3 siblings with severe, multi-organ RVCL involving the brain, eye, liver, and kidney, with onset of symptoms in the 4th and 5th decades of life (Fig. 1A-C). All of these siblings had the same *TREX1* variant (NM_033629.4): c.796G > T; p.E266X, encoding a C-terminally truncated TREX1 protein that leaves the catalytic domain entirely intact. This is similar to other truncated *TREX1* variants distal to the catalytic domain, which cause RVCL with 100% penetrance (Fig. 1D). We confirmed that the TREX1 E266X mutant is expressed in transfected 293T cells, and that the protein is truncated compared to WT TREX1 (Fig. 1E). In our large cohort of patients and relatives with RVCL, this *TREX1* variant represents 3.3% of patients (Fig. 1F). Using confocal microscopy, we confirmed that the TREX1 E266X protein is mislocalized throughout the cell including in the nucleus (Fig. 1G), a result that is consistent with previously reported RVCL-causing mutants [1, 3]. Indeed, aberrant nuclear localization of TREX1 drives DNA damage and disease in RVCL patients and in animal models of RVCL [5].

The patients’ mother had previously been diagnosed with retinal disease at age 41, which is the typical age of onset for RVCL. However, genetic testing of blood samples from both parents revealed that neither had the inherited disease-causing *TREX1* variant in their blood cells. Furthermore, several aspects of the maternal patient’s disease course are extremely unusual in RVCL for several reasons. At age 74, she still has no apparent extraretinal disease. Indeed, there have been no neurological symptoms, no renal impairment, and no liver disease, even though these manifestations of RVCL are universally present in her children and in other patients from our cohort beyond age 60. Skin biopsy revealed the *TREX1* c.796G > T (p.E266X) mutation, confirming maternal mosaicism. Additionally, she is the longest-lived patient in our large cohort of 120 patients and relatives with RVCL (Fig. 1F), and she also appears to be the longest-lived patient ever reported to have been diagnosed with RVCL [6]. Given the absence of multi-organ disease and her overall good health, we estimate that her life expectancy is likely to extend into the 9th or 10th decades of life.

These results demonstrate that three siblings with a standard presentation of RVCL inherited the disease-causing *TREX1* variant via maternal mosaicism. Interestingly, the mother developed eye disease at the same age as patients with germline RVCL-causing *TREX1* mutations, including her own children. This suggests that there may be a threshold percentage of cells within a particular tissue that is necessary to develop pathology, and yet age of onset appears to be the same in mosaic and germline cases.

**Fig. 2:**
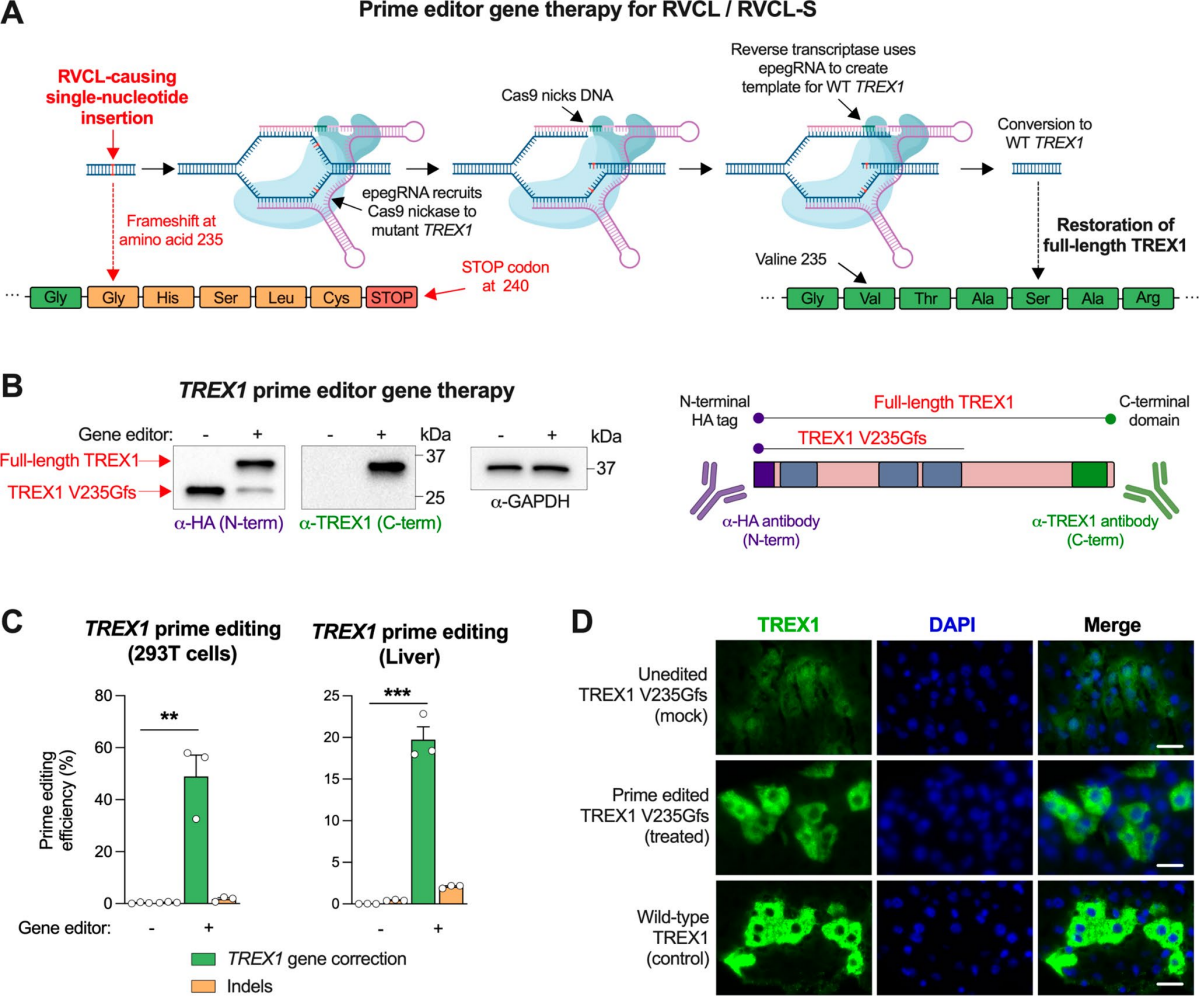
Prime-editing gene therapy converts the RVCL-causing TREX1 mutant protein to wild-type TREX1 in cultured cells and in mice. (**A**) Schematic of prime-editing gene therapy for TREX1 V235Gfs, Created in BioRender. Miner, J. (2024) https://BioRender.com/g08d573 (**B**) Western blot of TREX1 in 293T cells expressing TREX1 V235Gfs and transfected with the RVCL gene editor. After SDS-PAGE, membranes were probed with antibodies against the N-terminal HA tag (left), or an antibody against the C-terminus of TREX1 (middle), or GAPDH (right). On the right, we include diagram indicating the regions of TREX1 detected by antibodies to the N- and C-termini, created in BioRender. Miner, J. (2024) https://BioRender.com/t88w064. Data are representative of three independent experiments. (**C**) Prime editing efficiency in 293T cells (left) and livers of mice treated intravenously with AAV encoding the RVCL prime editor (right). For statistical analysis, the data represent the mean ± SEM of *n* = 3 samples pooled from three independent experiments and were analyzed by Student’s *t* test (^**^P < 0.01; ^***^P < 0.001). (**D**) Representative epifluorescence images of liver sections from unedited and AAV prime-edited LSL CAG-Cre TREX1 mice expressing HA-tagged TREX1 V235Gfs and WT TREX1 (control), expression was induced with daily tamoxifen injection for three or four days. Scale bar = 20 μm. Data in (**D**) are representative of two independent experiments

Since we discovered that mosaicism underlies a much milder, organ-limited disease phenotype with prolonged life expectancy, we reasoned that gene editing to create mosaicism in an adult might become a useful treatment option in the future, since it would permit correction of the disease-causing *TREX1* variant. However, traditional CRISPR/Cas9 deletion approaches to remove a mutant allele would not be useful for RVCL, since new insertions or deletions would create another frameshift at the same location, yielding a similarly pathogenic, truncated form of enzymatically active TREX1. Therefore, we developed a Cas9 prime editor strategy instead, which utilizes a Cas9 nickase and reverse transcriptase (RT), with a nicking guide RNA (ngRNA) and an engineered prime editing guide RNA (epegRNA) that serves as an RT template to generate the fully corrected *TREX1* DNA sequence for insertion into the genome (Fig. 2A). A major advantage of prime editing is that indels and other off-target effects are far less likely [8, 9]. To further minimize off-target effects and to prevent editing of WT *TREX1*, we designed a protospacer that utilizes a PAM site unique to the mutant *TREX1* variant. After extensive optimization over the course of years, we developed an efficient adeno-associated virus (AAV)-delivered prime editor that corrects the most common RVCL-causing mutation (TREX1 V235Gfs) in cell culture as well as in the livers of transgenic mice that express the human TREX1 V235Gfs mutant (Fig. 2B-C). Whereas RVCL-causing TREX1 mutants localize in the nucleus, the full-length WT form of TREX1 is excluded from the nucleus. Using immunofluorescence staining of TREX1 in the liver, we confirmed that treatment with the AAV-delivered gene therapy led to proper localization of TREX1 outside the nucleus, similar to localization of TREX1 in animals that express the cDNA encoding full-length WT TREX1 (Fig. 2D). In the future, we anticipate that further refinement of this technology and its cell type or tissue-specific delivery will be crucial.

## Discussion

We found that somatic mosaicism in *TREX1* can cause organ-limited RVCL with a normal lifespan, and we created a prime editor gene therapy that can correct the most common RVCL-causing TREX1 mutation. Since germline *TREX1* variants cause multi-organ disease and premature death in 100% of patients with RVCL, our results suggest that correction of the *TREX1* mutant with a gene therapy might prolong life and limit pathology by creating mosaicism in patients with germline *TREX1* variants.

Some have suggested that RVCL is related to systemic lupus erythematosus or that it might be an interferonopathy, which are diseases typically linked to the hematopoietic compartment [10, 11]. We do not exclude that systemic lupus may develop in rare cases of C-terminal TREX1 truncation. However, our mosaic patient with organ-specific RVCL had no detectable *TREX1* mutation in her blood, suggesting that expression of the disease-causing mutant in hematopoietic cells is not required for retinal vasculopathy. Since RVCL is associated with vascular-predominant processes in multiple organs, as well as histologically aberrant vascular endothelial architecture [6], we speculate that expression of the TREX1 mutant in endothelial cells, or perhaps in perivascular cells, likely drives the pathophysiology of RVCL. This would explain why we observed organ-limited but nevertheless severe disease in a patient with *TREX1* mosaicism.

Others have suggested that RVCL is a type I interferonopathy similar to AGS [10, 11]. Autoantibodies can develop in both of these diseases [12], and similar organs can be affected including the brain, liver, and kidney [1, 12]. However, AGS is characterized by severe systemic inflammation, and RVCL is not. Indeed, based on studies of large patient cohorts, and in contrast to AGS, patients with RVCL do not typically have elevated levels of type I IFN in their serum or elevated ISGs in their peripheral blood [5, 13]. This is consistent with the fact that AGS-causing *TREX1* variants are usually recessive and disrupt TREX1 exonuclease activity, while RVCL-causing variants are always dominant and leave the N-terminal exonuclease intact, thereby causing mislocalization of an active exonuclease [1]. RVCL is caused by heterozygous *TREX1* variants, which are not dominant negative, and therefore the patients still have one properly functioning full-length copy of TREX1, which is sufficient to prevent cGAS-STING activation, induction of type I IFN, and systemic inflammation [5]. Indeed, haploinsufficiency does not occur in AGS, as evidenced by the fact that the heterozygous parents and siblings of patients with AGS-causing loss-of-function *TREX1* variants are completely healthy [4].

Although patients with RVCL do not have systemic inflammation, we cannot exclude a role for local inflammation in RVCL. Indeed, TREX1 expression is regulated by cytokines [5, 14], and we previously found that inflammation up-regulates TREX1 and increases the DNA damage and pathology associated with an RVCL-causing mutant TREX1 in primary cells and in mice [5]. We speculate that there is a threshold expression level of TREX1, above which the C-terminally truncated mutant exhibits aberrant nuclear activity resulting in toxicity and pathology [5].

A key question arises regarding the literature’s confusion over whether RVCL is an interferonopathy or not. Our laboratory has worked in close collaboration with the laboratories of Osamu Onodera and Taisuke Kato at the Brain Research Institute at Niigata University (Japan) to unravel this conundrum [5]. Upon inducible expression of the RVCL-causing TREX1 mutant, we have observed cell death and senescence, leading to most surviving cells being TREX1-negative. After culturing cells for a couple of weeks, Western blot analysis confirms the presence of TREX1 mutant protein, though flow cytometric analysis shows that only a very small fraction  of cells actually express the mutant protein. This might explain the lower TREX1 mutant protein expression observed in cell culture, as we saw for the TREX1 E266X mutant in this study. While inefficient expression of a mutant may be sufficient for developing and testing a gene therapy in vitro, it does not adequately support the study of RVCL mechanisms for the following reasons: (A) *Selection events.* Relying solely on Western blot analyses could mislead researchers into thinking RVCL cell lines are suitable for studying the effects of truncated RVCL-causing TREX1. These TREX1 variants drive senescence and cell death due to TREX1-mediated DNA damage [5], highlighting the need for flow cytometric and quantitative microscopy to accurately characterize TREX1 expression in RVCL studies. (B) *Problems with exogenous TREX1 expression.* Some prior studies examining C-terminally truncated TREX1 were validated only by Western blot. Without using a second method to confirm expression levels in the entire population of cells, those studies likely did not appreciate the rapid selection events leading to predominantly TREX1-negative (or TREX1-low) cell populations. Consequently, while individual studies have reported a type I IFN signature in cell cultures or limited case reports of RVCL, large cohorts of patients with RVCL—including our own—do not exhibit such signatures [5, 13]. Thus, in cell culture studies, including some studies of patient cells or TREX1 knockout cells complemented with mutant protein, the RVCL mutant-mediated selection and associated TREX1 deficiency has potential to create the illusion of an IFN signature associated with RVCL.

There are currently no FDA-approved medications for the treatment of RVCL. In the past, chemotherapy was proposed as a treatment and tested in these patients based on the idea that there might be underlying IFN signatures or glycan abnormalities in RVCL [11, 15], but we now know that chemotherapy is likely to be particularly damaging in patients with RVCL, since heightened vulnerability to chemotherapy is a hallmark of TREX1-mediated DNA damage and immune dysregulation [5]. Indeed, cells and animals with RVCL-causing TREX1 mutants exhibit high levels of DNA damage in response to multiple chemotherapies, interferons, and pro-inflammatory cytokines [5]. Thus, there is a critical need for targeted therapies for RVCL, including gene therapies. Our prime editor gene therapy corrects the most common RVCL-causing mutation in mice, but further refinement of this technology is necessary for potential therapeutic use. For instance, repeated dosing of the gene therapies to increase editing efficiency remains a challenge in the gene therapy field, since therapies delivered by viral vectors can induce an immune response that prevents multiple administrations.

Our patient with *TREX1* mosaicism would not have been diagnosed with RVCL had it not been for germline transmission to her progeny. This is an important proof-of-principle for monogenic human diseases, since *TREX1* mosaicism in this case was identified only because of germline transmission. Indeed, many patients with unique disease phenotypes may have organ-specific pathology due to somatic mosaicism that did not undergo germline transmission to progeny. Thus, the continued expansion of personalized medicine-based approaches for diagnosing and treating patients will require the development of organ-specific genetic testing and therapies to effectively identify and treat diseases caused by somatic mosaicism.

## Methods

### Approvals

Protocols for mouse studies were approved by the Institutional Animal Care and Use Committee (IACUC) at Penn. Patients were enrolled in an institutional review board (IRB)-approved longitudinal clinical study (REVEAL study) at Penn.

### Mice

Human LSL TREX1 V235Gfs and LSL WT TREX1 transgenic mice were generated by the Miner laboratory at Penn and were previously described [5]. These animals have a CAG promoter and lox-stop-lop sequence preceding either the human WT TREX1 cDNA or TREX1 V235Gfs cDNA in the ROSA26 locus. Transgenic human TREX1 animals were crossed to tamoxifen-inducible CAG-Cre transgenic mice (Jax 004682). Mice were housed in pathogen-free facilities at Penn and fed a standard diet *ad libitum*. Experiments were performed using mice of both sexes at ages 8–12 weeks.

### Cell Culture Studies of TREX1

Of note, the TREX1 V235Gfs mutant is toxic to cells, requiring flow cytometric screening to confirm expression. This is an absolute requirement for proper studies of RVCL-causing *TREX1* variants, since only a small percentage of cells can tolerate expression of the RVCL-causing mutant.

For in vitro development of the gene therapy and localization studies, we utilized 293T cells cultured in Dulbecco’s modified eagle medium (DMEM; Gibco, 11995081) supplemented with 10% fetal bovine serum (FBS), 2 mM L-GlutaMAX (Gibco, 35050061), 1X non-essential amino acids (Gibco, 11140050), 1 mM sodium pyruvate (Gibco, 11360070), 10 mM HEPES (Gibco, 15630080), 100 U/mL penicillin, 100 μg/mL streptomycin (Gibco, 15140122) at 37˚C and 5% CO_2_. To examine protein expression and to develop gene therapies, the WT TREX1 and TREX1 V235Gfs cDNAs were synthesized by Genscript, (Piscataway, NJ), including an N-terminal HA tag, and cloned into the multiple cloning site of the doxycycline-inducible pTRIPZ vector (AgeI/MluI). To produce pTRIPZ-TREX1 E266X, pTRIPZ-WT TREX1 was mutagenized via PCR with the following primers: 5’ GCCTTGGATAGAGCAGGGGTACCA 3' and 5’ TGGTACCCCTGCTCTATCCAAGGC 3'. Linear PCR amplicons were treated with DpnI for 3 hours at 37 °C and used to transform XL1-Blue chemically competent cells. The sequences of all plasmids were confirmed using Sanger sequencing. To produce lentivirus, 293T cells were seeded at a density of 5 × 10^5^ cells per well in a 6-well plate and were transfected with a 4:2:1 ratio of pTRIPZ: psPAX2:VSVG for a total DNA mass of 2.5 µg/well. Cells were incubated for 72 hours. After incubation, supernatants were filtered through 0.45 μm syringe filters, aliquoted and stored at -80 °C.

To create a stable cell line with inducible expression of WT TREX1 or TREX1 E266X, 293T cells were seeded at a density of 5 × 10^5^ cells per well in the wells of a 6 well plate. pTRIPZ-TREX1 lentiviral supernatants were diluted 1:5 in DMEM supplemented with 8 µg/mL polybrene and incubated for 72 hours. Following incubation, cells were reseeded in 10 cm dishes in DMEM supplemented with 1.5 µg/mL puromycin and cultured for 7 days. TREX1 expression was induced by culturing cells in DMEM with doxycycline (500 ng/mL) for 2 days. Longer periods of TREX1 induction induces selection events in cells expressing the RVCL-causing TREX1 variants, since these mutants are now well-known to cause DNA damage in cultured cells and in animals.

### SDS PAGE and Western Blotting

Liver samples were homogenized in RIPA buffer (CST, 9806) with protease inhibitors (Sigma, 11836170001). Cultured cells were lysed in RIPA buffer with protease inhibitors (80 µL per 1 million cells). Then, 70 µL of cell lysates were mixed with 35 µL of 6x Laemmli sample buffer with 5% β-mercaptoethanol and boiled at 95˚C for 5 minutes. Samples were then loaded onto a 10% Tris/glycine SDS-PAGE gel (Biorad) and transferred to PVDF membranes. Membranes were blocked for 1 hour at room temperature with 3% BSA in TBST and incubated with primary antibodies against TREX1 (CST, 15107, 1:1,000), HA (CST, 3724, 1:1000) or GAPDH (CST, 2118, 1:1,000) overnight. For cell culture studies of WT TREX1 and TREX1 E266X, an N-terminal anti-HA antibody was used to probe membranes. Secondary staining was performed using horseradish peroxidase-conjugated antibodies against rabbit (CST, 7076, 1:10,000) or mouse (CST, 7074, 1:10,000) IgG. Blots were developed using Pierce ECL Western blotting substrate (Thermo, 32106) and scanned with a Bio-Rad XRS + gel imaging system.

### Confocal Microscopy Studies

For confocal microscopy analysis of TREX1 localization, 293T cells with inducibly expressed HA-tagged WT TREX1 or TREX1 E266X were cultured for 2 days on poly-L-lysine-coated coverslips in complete media with doxycycline (500 ng/mL). Then cells underwent fixation with 4% paraformaldehyde for 15 minutes at room temperature. Cells were then permeabilized and blocked with blocking buffer (5% donkey serum in PBS with 0.3% Triton X-100). The cells were immunostained overnight with anti-HA antibody (CST 2367 S, 1:10000) in blocking buffer at 4˚C. Secondary staining was performed with AF647- or AF488-conjugated anti-rabbit secondary antibody for 1 hour at room temperature. Nuclei were counterstained with diamidino-2-phenylindole dihydrochloride (DAPI) and mounted in Prolong Gold anti-fade mounting medium. Images were acquired on the Leico TCS SP8 WLL Confocal and were analyzed in ImageJ.

### Immunofluorescence Analysis of TREX1 in Mouse Livers

Liver tissue was excised from euthanized mice and then flash frozen in OCT-embedding media on dry ice. OCT-blocked livers were sectioned to a thickness of 8 μm by the University of Pennsylvania Molecular Pathology and Imaging Core. Frozen sections were allowed to thaw for 5 minutes at room temperature before fixation with 3% paraformaldehyde for 15 minutes at room temperature. Tissue sections were washed with 1X TBST (CST) and blocked with 1X TBST supplemented with 10% normal donkey serum. Sections were incubated with anti-HA (CST, 3724) overnight at 4 °C in a humidified chamber and visualized with fluorescent secondary antibody donkey anti-rabbit AF488 (Invitrogen). Nuclei were stained with DAPI and then coverslips applied with Prolong Gold anti-fade mounting medium. Epifluorescence images were acquired on an EVOS M5000 microscope and analyzed using FIJI software.

### Screening of epegRNAs and Deep Sequencing Analysis

We began to develop a gene therapy by designing hundreds of potential prime editor gRNA (pegRNA) sequences followed by engineered pegRNA (epegRNA) sequences. Construction of epegRNA and sgRNA expression plasmids was performed as described previously [16]. Briefly, epegRNA and sgRNA oligonucleotides were designed with the assistance of prime design software [17] and synthesized by IDT. Oligonucleotides were annealed and cloned into epegRNA acceptor plasmid pU6-tevopreq1-GG-acceptor (Addgene, 174038) and sgRNA acceptor plasmid pU6-pegRNA-GG-acceptor (Addgene, 132777) digested with BsaI and assembled via Gibson assembly using T4 DNA ligase. At these initial stages, we screened 6 protospacers with 19 unique reverse transcription template (RTT) sequences. Thus, many unique epegRNAs were screened as potential prime editors of the TREX1 V235Gfs mutant. Transfected 293T cells were trypsinized and washed with 1 mL DPBS. DNA was extracted using the Qiagen DNeasy extraction kit and eluted in 100 μL elution buffer AE. The TREX1 V235Gfs cDNA was PCR amplified with the following PCR primers 5’ ACACTCTTTCCCTACACGACGCTCTTCCGATCTGTGATGTCCTGGCCCTGCT 3' and 5’ GACTGGAGTTCAGACGTGTGCTCTTCCGATCTCCCTTCGTCTGACGTGGCAGC 3' (Illumina adaptor sequences are underscored). For analysis of liver tissue from AAV-treated mice, livers were excised, and DNA was extracted using the Qiagen DNeasy extraction kit and eluted in 200 µL elution buffer AE. PCR amplicons were generated using the following PCR primers (Illumina adaptor sequences underscored) 5’ ACACTCTTTCCCTACACGACGCTCTTCCGATCTGTGATGTCCTGGCCCTGCT 3’ and 5’ GACTGGAGTTCAGACGTGTGCTCTTCCGATCTGATAGGCAGCCTGCACC 3’ NGS Amplicon sequencing was performed by Azenta, and editing efficiency was determined using the Crispresso2 web tool [18]. Editing efficiency was determined by dividing unmodified HDR reads/total aligned reads and expressed as a percentage. Indels were calculated by the sum of the modified reference and HDR reads/total aligned reads and expressed as a percentage.

### Adeno-Associated Virus (AAV)

For construction of AAV genome plasmid encoding the epegRNA and sgRNA oligonucleotides were designed to PCR amplify the human U6 promoter and tevopreq motif, a second PCR was performed amplifying the desired epegRNA cassette. A third PCR reaction was performed amplifying the sgRNA cassette and mouse U6 promoter sequence. These DNA fragments were assembled with PCR amplicons encoding the AAV vector backbone derived from V3em-Cterm-PE2MAX-ΔRnaseH-dualU6 (Addgene, 198735) using Nebuilder HIFI assembly master mix (NEB) according to manufacturer’s instructions. The epegRNA sequence was: ACCATCAGGCCCATGTATGGGTTTTAGAGCTAGAAATAGCAAGTTAAAATAAGGCTAGTCCGTTATCAACTTGAAAAAGTGGCACCGAGTCGGTGCAGAGGCTGTGACCCCATACATGGGCCTGATCGCGGTTCTATCTAGTTACGCGTTAAACCAACTAGAA. The sgRNA sequence used in cells was: GAGGCTGTGACgccATACATGTTTTAGAGCTAGAAATAGCAAGTTAAAATAAGGCTAGTCCGTTATCAACTTGAAAAAGTGGCACCGAGTCGGTGC. The sgRNA sequence used in mice was: GAGGCTGTGACCCCATACATGTTTTAGAGCTAGAAATAGCAAGTTAAAATAAGGCTAGTCCGTTATCAACTTGAAAAAGTGGCACCGAGTCGGTGC. Prime editor AAV plasmids were transferred to Packgene Biotech for packaging with AAV serotype 8.

### Gene Editing in mice

CAG-Cre LSL TREX1 V235Gfs mice were intravenously injected (via the retroorbital plexus) with 1 × 10^12^ genome copies (GCs) of both N- and C-terminal AAV prime editors (for a total of 2 × 10^12^ GCs). TREX1 expression was induced on day 18 after AAV transduction by daily intraperitoneal injection of tamoxifen (75 mg/kg) for three days. Timing was based on the fact that we have observed optimal expression of TREX1 after 4 days of induction. Liver samples were collected on day 22, andtiming of harvest after AAV administration was based on previous work using AAV gene therapies [19].

### Statistics

Statistical analyses are indicated in the figure legends and were conducted in Prism (GraphPad) Version 10.4.0.

## Data Availability

No datasets were generated or analysed during the current study.

## References

[1] Rice GI, Rodero MP, Crow YJ. Human Disease Phenotypes Associated with mutations in TREX1. J Clin Immunol. 2015;35(3):235–43. 25731743 10.1007/s10875-015-0147-3

[2] Rice G, Newman WG, Dean J, Patrick T, Parmar R, Flintoff K, et al. Heterozygous mutations in TREX1 cause familial chilblain lupus and dominant Aicardi-Goutieres syndrome. Am J Hum Genet. 2007;80(4):811–5. 17357087 10.1086/513443 PMC1852703

[3] Richards A, van den Maagdenberg AMJM, Jen JC, Kavanagh D, Bertram P, Spitzer D, et al. C-terminal truncations in human 3′-5′ DNA exonuclease TREX1 cause autosomal dominant retinal vasculopathy with cerebral leukodystrophy. Nat Genet. 2007;39(9):1068–70. 17660820 10.1038/ng2082

[4] Crow YJ, Hayward BE, Parmar R, Robins P, Leitch A, Ali M, et al. Mutations in the gene encoding the 3′-5′ DNA exonuclease TREX1 cause Aicardi-Goutières syndrome at the AGS1 locus. Nat Genet. 2006;38(8):917–20. 16845398 10.1038/ng1845

[5] Chauvin SD, Ando S, Holley JA, Sugie A, Zhao FR, Poddar S, et al. Inherited C-terminal TREX1 variants disrupt homology-directed repair to cause senescence and DNA damage phenotypes in Drosophila, mice, and humans. Nat Commun. 2024;15(1):4696. 38824133 10.1038/s41467-024-49066-7 PMC11144269

[6] Stam AH, Kothari PH, Shaikh A, Gschwendter A, Jen JC, Hodgkinson S, et al. Retinal vasculopathy with cerebral leukoencephalopathy and systemic manifestations. Brain. 2016;139(11):2909–22. 27604306 10.1093/brain/aww217 PMC5091044

[7] Miner JJ, Fitzgerald KA. A path towards personalized medicine for autoinflammatory and related diseases. Nat Rev Rheumatol. 2023;19(3):182–9. 36750685 10.1038/s41584-022-00904-2 PMC9904876

[8] Newby GA, Liu DR. In vivo somatic cell base editing and prime editing. Mol Ther. 2021;29(11):3107–24. 34509669 10.1016/j.ymthe.2021.09.002 PMC8571176

[9] Anzalone AV, Koblan LW, Liu DR. Genome editing with CRISPR-Cas nucleases, base editors, transposases and prime editors. Nat Biotechnol. 2020;38(7):824–44. 32572269 10.1038/s41587-020-0561-9

[10] Wolf C, Rapp A, Berndt N, Staroske W, Schuster M, Dobrick-Mattheuer M, et al. RPA and Rad51 constitute a cell intrinsic mechanism to protect the cytosol from self DNA. Nat Commun. 2016;7:11752. 27230542 10.1038/ncomms11752 PMC4895045

[11] Hasan M, Fermaintt CS, Gao N, Sakai T, Miyazaki T, Jiang S, et al. Cytosolic nuclease TREX1 regulates oligosaccharyltransferase activity Independent of nuclease activity to suppress Immune activation. Immunity. 2015;43(3):463–74. 26320659 10.1016/j.immuni.2015.07.022 PMC4575271

[12] Peixoto de Barcelos I, Jan AK, Modesti N, Woidill S, Gavazzi F, Isaacs D, et al. Systemic complications of Aicardi Goutières syndrome using real-world data. Mol Genet Metab. 2024;143(1):108578. 39332260 10.1016/j.ymgme.2024.108578 PMC12302025

[13] Rodero MP, Decalf J, Bondet V, Hunt D, Rice GI, Werneke S, et al. Detection of interferon alpha protein reveals differential levels and cellular sources in disease. J Exp Med. 2017;214(5):1547–55. 28420733 10.1084/jem.20161451 PMC5413335

[14] Serra M, Forcales S-V, Pereira-Lopes S, Lloberas J, Celada A. Characterization of Trex1 induction by IFN-γ in Murine macrophages. J Immunol. 2011;186(4):2299–308. 21239708 10.4049/jimmunol.1002364

[15] Atkinson JP. Aclarubicin for the Treatment of Retinal Vasculopathy With Cerebral Leukodystrophy (RVCL). 2020; https://clinicaltrials.gov/ct2/show/NCT02723448

[16] Doman JL, Sousa AA, Randolph PB, Chen PJ, Liu DR. Designing and executing prime editing experiments in mammalian cells. Nat Protoc. 2022;17(11):2431–68. 35941224 10.1038/s41596-022-00724-4 PMC9799714

[17] Hsu JY, Grünewald J, Szalay R, Shih J, Anzalone AV, Lam KC, et al. PrimeDesign software for rapid and simplified design of prime editing guide RNAs. Nat Commun. 2021;12(1):1034. 33589617 10.1038/s41467-021-21337-7 PMC7884779

[18] Clement K, Rees H, Canver MC, Gehrke JM, Farouni R, Hsu JY, et al. CRISPResso2 provides accurate and rapid genome editing sequence analysis. Nat Biotechnol. 2019;37(3):224–6. 30809026 10.1038/s41587-019-0032-3 PMC6533916

[19] Davis JR, Banskota S, Levy JM, Newby GA, Wang X, Anzalone AV, et al. Efficient prime editing in mouse brain, liver and heart with dual AAVs. Nat Biotechnol. 2024;42(2):253–64. 37142705 10.1038/s41587-023-01758-z PMC10869272

